# Photodegradation of Ibuprofen, Cetirizine, and Naproxen by PAN-MWCNT/TiO_2_–NH_2_ nanofiber membrane under UV light irradiation

**DOI:** 10.1186/s12302-018-0177-6

**Published:** 2018-12-03

**Authors:** Alaa Mohamed, Ahmed Salama, Walaa S. Nasser, Abdusalam Uheida

**Affiliations:** 1 Egypt Nanotechnology Center, EGNC, Cairo University, Giza, 12613 Egypt; 2 Production Engineering and Printing Technology Department, Akhbar El Yom Academy, Giza, 12655 Egypt; 3 Department of Production Engineering and Manufacturing Technology, Modern Academy for Engineering and Technology In Maadi, Cairo, Egypt; 4 Research Institute of Medical Entomology, Giza, 12611 Egypt; 5 Department of Applied Physics, School of Engineering Sciences, Royal Institute of Technology (KTH), 16440 Kista, Stockholm Sweden

**Keywords:** Photocatalytic, Cetirizine, Naproxen, Ibuprofen, Composite nanofibers, UV-light

## Abstract

**Background:**

In this study, the photodegradation of three pharmaceuticals, namely Ibuprofen (IBP), Naproxen (NPX), and Cetirizine (CIZ) in aqueous media was investigated under UV irradiation. The photocatalyst used in this work consists of surface functionalized titanium dioxide (TiO_2_–NH_2_) nanoparticles grafted into Polyacrylonitrile (PAN)/multi-walled carbon nanotube composite nanofibers. Surface modification of the fabricated composite nanofibers was illustrated using XRD, FTIR, and SEM analyses.

**Results:**

Sets of experiments were performed to study the effect of pharmaceuticals initial concentration (5–50 mg/L), solution pH (2–9), and irradiation time on the degradation efficiency. The results demonstrated that more than 99% degradation efficiency was obtained for IBP, CIZ, and NPX within 120, 40, and 25 min, respectively.

**Conclusions:**

Comparatively, the photocatalytic degradation of pharmaceuticals using PAN-CNT/TiO_2_–NH_2_ composite nanofibers was much more efficient than with PAN/TiO_2_–NH_2_ composite nanofibers.

## Introduction

Nowadays, the world recognizes the importance of the continuous development on the area of synthesis and production of a variety of pharmaceutical drugs for both humans and animals. Large scales of different chemical compounds are used as medicinal products. These compounds classified as emerging pollutants, while their essential passage into the environment is pharmaceutical industries, excretory products of medically treated humans and animals, which followed by their inefficient removal in wastewater treatment plants [1, 2]. Also they enter to the environment after inappropriate disposal of expired pharmaceutical products in the sewage system or in the garbage. The extended usage of dangerous pharmaceuticals is followed by an increased pollution of ground, surface, and drinking water by these compounds [3, 4].

The most considerably detected groups of pharmaceuticals in the environment are Non-steroidal anti-inflammatory drugs (NSAIDs). These are one of the widely available drugs in the world. NSAID group have a main common characteristic, which is the carboxylic aryl acid moiety, which provides their acidic properties. Ibuprofen (IBP) belongs to this family of pharmaceuticals, which is an analgesic drug highly used for the medicament of myoskeletal injuries, rheumatoid arthritis, and fever [5]. A large number of NSAIDs are widely existing in surface water, these contaminations are highly problematic because the surface waters are main resources used for drinking water production. Choina et al. [6], studied the degradation of the IBP and the results indicated that the photocatalytic treatment with titania catalyst leads to rapid mineralization of ibuprofen. NPX also belongs to the non-steroidal anti-inflammatory drug (NSAID) [7]. In addition, NPX is used as an antipyretic and analgesic drug for the treatment of rheumatoid arthritis. In addition, it is also used as veterinary medicine [8]. Ray et al. [9], studied the degradation of the NPX over AgBr-α-NiMoO_4_ composite photocatalyst and the results show that the composite photocatalyst degraded NPX drug rapidly within 20 min under visible light irradiation. The photocatalytic performances of composite stayed efficient up to fifth cycle, and this indicates to the excellent stability [10, 11]. Moreover, CIZ append to the piperazine class of second generation antihistamines used in the treatment of allergies, hay fever, angioedema, and urticarial and causes various side effects such as dry mouth, sleepiness, sedation, fatigue, fever, stomach upset, and blurred vision when ingested accidentally to the human body [12]. The degradation of CIZ has not reported neither by enzymatic method nor by ultrasound assisted enzymatic method. Rahul et al. [13], studied the degradation of the CIZ using a novel technique of laccase enzyme as a catalyst under the influence of ultrasound irradiation. The results show that the degradation of CIZ achieved was 91% in comparison of ultrasound assisted enzymatic degradation with conventional at a shorter time.

The most conventional treatment processes applied at domestic wastewater treatment plants fail to remove completely pharmaceutical substances. Therefore, the treatment of the polluted water requires the application of effective techniques such as Advanced Oxidation Processes (AOPs) [14, 15]. Among AOPs, photocatalysis is a very important and attractive process. Heterogeneous photocatalytic water treatment has a highly special interest since it doesn’t require the use of any additional chemicals [16, 17]. The photocatalytic processes mainly use semiconducting material catalysts such as (TiO_2_, Fe_2_O_3_, ZnO) under light exposure (UV-light or sunlight) to degrade the organic and inorganic contaminants. Most of the researches are now focusing on the photocatalytic techniques using composite nanofibers [18, 19]. Many semiconducting catalysts can be used, but Titania (TiO_2_) is the most common semiconducting catalyst used due to its cheap cost combined with a high photocatalytic activity, nonhazardous compound and eco-friendly [20, 21]. The application of Titania catalyst is most attractive for effective photocatalytic degradation of drugs and other harmful organic pollutants assisted by UV–Vis radiation. In this work, PAN nanofibers were used as substrate for holding the photocatalyst material. On the other hand, MWCNTs was used to improve the tensile strength, young’s modulus, and chemical resistance of the nanofiber due to their high physical, and chemical properties [22–24]. In addition, CNT may increases the photocatalytic activity of the process due to its electron donor properties. Moreover, it offers the possibility of complete abatement of drugs and can be recovered and re-used [25, 26]. Therefore, the aim of this work was to investigate the photodegradation of IBP, NPX, and CIZ in aqueous media by means of composite nanofibers (PAN-MWCNT/TiO_2_–NH_2_) under UV irradiation. The effect of operating conditions such as initial drug concentration, and solution pH were evaluated.

## Experimental

### Material

TiO_2_ Degussa P-25, 3-aminopropyltriethoxysilane (APTES), Glutaraldehyde (GA), polyacrylonitrile, PAN (MW = 150,000); dimethylformamide (DMF), hydrochloric acid (HCI), sodium hydroxide (NaOH), Ibuprofen, Naproxen, and Cetirizine were purchased from sigma Aldrich and used as received. Multi-walled carbon nanotubes, MWCNTs (purity 95 wt%; diameter: 10–40 nm; length: 20 μm; specific surface area 460 m^2^/g) were synthesized and the procedure is described elsewhere [27, 28]. The chemical structures of the pharmaceuticals are presented in Table 1.

**Table 1:** Usage and chemical structure for Ibuprofen, Naproxen, and Cetirizine

Pharmaceuticals	Usage	Chemical structure
Ibuprofen	Analgesic, anti-inflammatory	
Naproxen	Analgesic, anti-inflammatory	
Cetirizine	Anti-allergic	

### Composite nanofibers preparation

A 10 wt% solution of PAN powder in DMF was prepared by mixing 1 g PAN with 9 mL DMF for 4 h at 50 °C. In parallel, 3 wt% surface activated MWCNTs were dispersed in DMF; the dispersion was stirred for 15 min, and then sonicated for 30 min. PAN solution was then added to the MWCNTs. The mixture was magnetically stirred for 15 min and then sonicated for 3 h. The electrospinning process was conducted at room temperature using a voltage of 25 kV, flow rate of 0.5 ml/h, and distance from needle tip to collector was 15 cm [29]. The electrospun PAN/MWCNT nanofibers were dried in vacuum over night to remove the excess amount of solvent. The electrospun composite nanofibers was cross-linked to TiO_2_–NH_2_ NPs. The composite nanofibers was then immersed in the crosslinking medium, which consisting of 100 mL distilled water and 2.5 wt% GA and placed in a mechanical shaker for 24 h at the room temperature. After the crosslinking process the composite nanofibers was washed with deionized water. The surface functionalization TiO_2_–NH_2_ NPs was suspended in 5 mL deionized water, afterward sonicated for 2 h and then added to the cross-linked composite nanofibers and mixed using mechanical shaker for 24 h. The PAN-CNT/TiO_2_–NH_2_ composite nanofibers was finally washed with deionized water and dried in air at the room temperature.

### Photocatalyst characterization

The morphology of the composites nanofibers was examined using Scanning Electron Microscopy (SEM, Gemini Zeiss-Ultra 55). Fourier transform infrared spectroscopy (FTIR, Nicolet iS10) was used to confirm the presence of amino groups on the TiO_2_ NPs attached to the surface of the composite nanofibers. The crystal phases of the composite nanofibers were evaluated by X-ray diffractometry (XRD, Bruker D8) using Cu Kα radiation (*λ*  =  1.5406 Å). The concentration of IBP, NPX, and CTZ in the solution was measured using UV–Vis/NIR spectrophotometer (model LAMBDA 750, Perkin Elmer) at maximum absorption wavelength (*λ*_max_) 222 nm, 229 nm and 232 nm for IBP, CTZ, and NPX, respectively.

### Degradation experiments

A stock solution of 100 mg/L of each pharmaceutical was prepared by addition of the appropriate amount of the pharmaceutical to 500 mL deionized water. Different drugs concentration (5, 10, 20, 30, and 50 mg/L) at different pH starting from 2 to 9 were prepared using the stock solution. For detection and quantification purposes, the range of concentrations in this study is higher than those typically detected in the environment. Using a UV-A lamp (315–400 nm) of 40 Watts for the UV-light irradiation which, suitable for the photocatalytic process especially whose work with polymer based composite nanofibers. The photocatalytic activity of PAN nanofibers, and composite nanofibers of PAN/TiO_2_–NH_2_ and PAN-MWCNT/TiO_2_–NH_2_ was studied by recording the degradation of IBP, NPX, and CIZ using UV–Vis spectrophotometer. The photocatalysis experiments were performed by placing the composite nanofibers mat in a column (2 cm × 30 cm) and the drug solution of 100 mL was circulated at a flow rate of 7 mL/min under UV-light irradiation. 5 mL of the drug solution were taken at fixed intervals of time. The solution pH was adjusted between 2 and 9 by the addition of HCl or NaOH. The pH was measured using a pH meter (WTW pH-330, Germany). After each experiment, the composite nanofibers were washed many times using deionized water to be able to reuse. All experiments were duplicated to assure the consistency and reproducibility of the results. The photodegradation efficiency was calculated according to the following equation:

1 $${\text{Degradation efficiency }}\left( \% \right) \, = \frac{{C_{i} - C_{t} }}{{C_{i} }} \times { 1}00$$

where, *C*_*i*_ (mg/L) is the initial drug concentration and *C*_*t*_ (mg/L) is the drug concentration at time (*t*).

## Results and discussion

### Photodegradation performance

#### Effect of catalyst amount

**Fig. 1:**
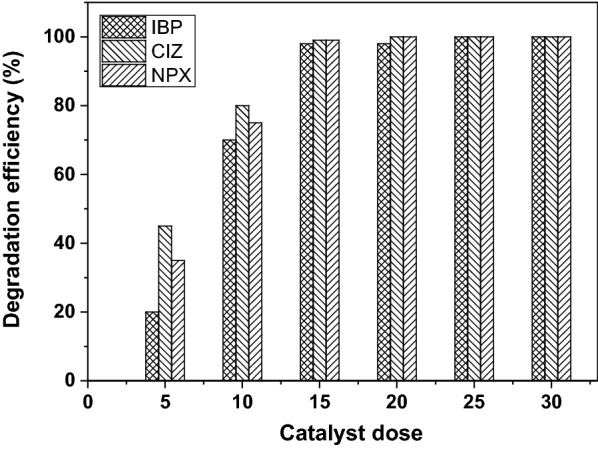
Effect of photocatalyst amount on the photodegradation efficiency of IBP, NPX, and CTZ under UV-light irradiation using PAN-MWCNT/TiO_2_–NH_2_ composites nanofibers. The drug solution = 5 mg/L, pH = 2, and total volume of 100 mL, irradiation time (IBP = 120 min, NPX = 40 min, CIZ = 25 min)

In order to determine the effect of the photocatalyst amount, a set of experiments were performed by varying the amount of the catalyst from 5 to 30 mg in a total volume of 100 mL and drug concentration of 5 mg/L at pH = 2. Figure 1 illustrates the effect of the photocatalyst amount on the photodegradation efficiency of pharmaceuticals. The results obtained demonstrated that photocatalytic degradation performance was substantially improved with increasing the amount of the photocatalyst and reached a plateau at a dosage of 15 mg (PAN-MWCNT/TiO_2_–NH_2_). This may be due to the increased number of active sites on PAN-MWCNT/TiO_2_–NH_2_ surface. Consequently, the increased generation of electron–hole pairs on the surface of the catalyst subsequently leading to higher amounts of reactive hydroxyl radicals that can be attributed to the better degradation observed. The increase in MWCNT/TiO_2_–NH_2_ loading provides more binding sites for substrate molecules to adsorb on the catalyst surface. In this study the fabricated composite nanofibers with 75% TiO_2_–NH_2_ NPs and 25% of MWCNT was used in all experiments.

#### Effect of exposure time

**Fig. 2:**
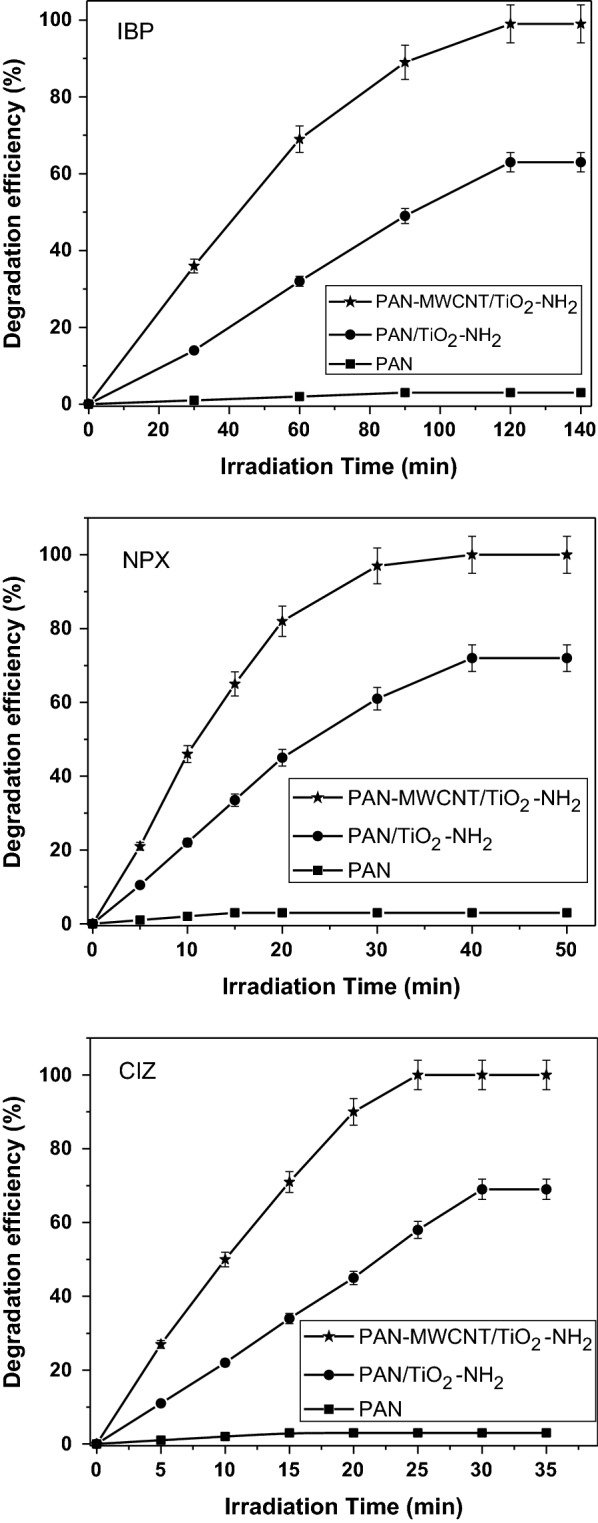
Photodegradation efficiency of IBP, NPX, and CIZ as a function of UV-light exposure time. The drug concentration = 5 mg/L, total volume = 100 mL, and pH = 2

The photocatalytic degradation was carried under controlled condition using PAN nanofiber, PAN/TiO_2_–NH_2_, and PAN-MWCNT/TiO_2_–NH_2_ composite nanofibers to illustrate the effect of the catalyst (TiO_2_–NH_2_ and MWCNT) on the composite nanofiber. Figure 2 shows the influence of exposure time on the photodegradation efficiency of IBP, NPX, and CIZ under UV-light. As can be observed from the results, the PAN-MWCNT/TiO_2_–NH_2_ composite nanofibers showed the highest degradation efficiency for all tested contaminates, which is attributed to the high surface to mass ratio for the composite nanofibers. Additionally, CNTs can effectively generate a number of free electrons and holes, which leads to acceleration of the photocatalytic reaction to enhance the photocatalytic activity. The complete degradation was observed at 120 min for IBP, 40 min for NPX, and 25 min for CIZ.

#### Effect of drug initial concentration

**Fig. 3:**
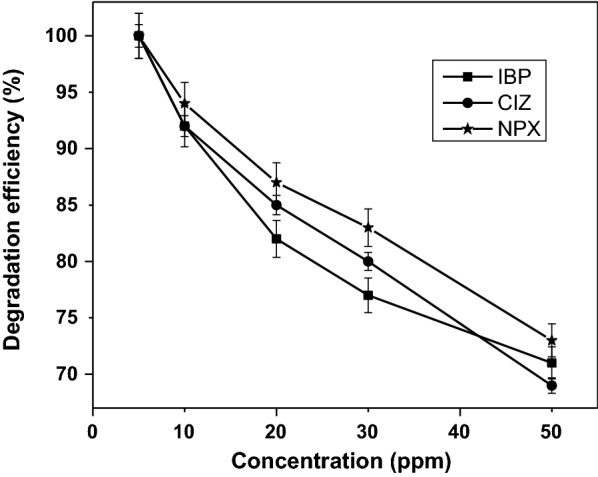
Effect of the initial drugs concentration on the degradation efficiency under UV-light irradiation at pH 2, drug solution volume of 100 mL, and irradiation time of IBP = 120 min, NPX = 40 min, and CIZ = 25 min

The photocatalytic degradation efficiency of a certain drug depends on its concentration, nature and on the presence of other existing compounds in the water matrix. Figure 3 shows the effect of IBP, NPX, and CIZ initial concentration on their photodegradation efficiency. The degradation efficiency was studied at initial concentrations of 5, 10, 20, 30, and 50 mg/L. The results obtained indicated that the photodegradation efficiency of IBP, NPX, and CIZ decreased from 100 to 70% as their initial concentration increased from 5 to 50 mg/L. This may be attributed to the decrease of hydroxyl radicals on the catalyst surface as the initial drug concentration is increased. As more drug molecules are adsorbed on the surface of the photocatalyst (MWCNT/TiO_2_–NH_2_), less active sites for the adsorption of hydroxyl radicals will be available. Hence, large amounts of adsorbed pharmaceuticals would have an inhibitory influence on the reaction between pharmaceuticals molecules and hydroxyl radicals due to the lack of any direct contact between them. On the other hand, most of the UV-light is absorbed by as the drug molecules as their concentration is increased, and the photons do not reach the surface of photocatalyst to generate hydroxyl radicals [30]. In addition, the photocatalytic degradation reaction will be deactivated due to the saturated surface of the photocatalyst, which caused by the high concentration of the drug.

#### The Effect of the pH of the solution

**Fig. 4:**
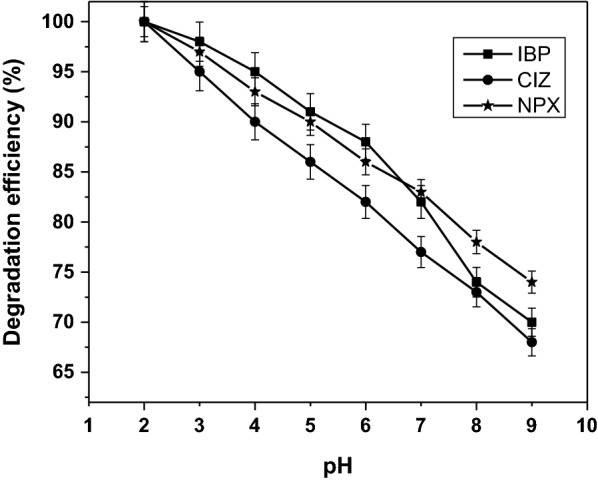
Effect of the pH on the degradation efficiency for IBP, NPX, and CIZ at initial concentration of 5 mg/L, drug solution volume of 100 mL, and irradiation time of IBP = 120 min, NPX = 40 min, and CIZ = 25 min

The photocatalytic abatement of IBP, NPX, and CIZ over TiO_2_–NH_2_ is significantly improved with decreasing pH value. The effect of pH on the degradation efficiency of pharmaceuticals drugs was studied by varying the solution pH from 2 to 9 at fixed catalyst amount of 15 mg and UV-light irradiation time of 120, 40, and 25 min for IBP, NPX, and CIZ, respectively. Figure 4 shows that the acidic conditions showed higher degradation efficiency and the maximum was obtained at pH 2. In a weak acidic medium, the pharmaceutical drugs are electrically neutral and exist in molecular form, while TiO_2_–NH_2_ is protonated and electropositive [31, 32]. The efficiency of the process increases by the formation of hydroxyl radicals that formed when the hydroxide ions interact with the positive holes at low pH [33], which attributed due to the electrostatic interactions between the positive catalyst surface and the pharmaceuticals drug anions, leading to strong adsorption of the pharmaceuticals drug anions on the metal oxide support. However, in strong acidic media, TiO_2_–NH_2_ is electropositive and the concentration of H^+^ is excessive, which might lead to the reductions of OH·. As a result, the degradation efficiency of the pharmaceutical drugs was decreased. In neutral and alkaline media, the pharmaceutical drugs are electronegative due to ionization. Therefore, it can repel electronegative TiO_2_–NH_2_ and OH^−^ [34].

#### Nanocomposite stability

**Fig. 5:**
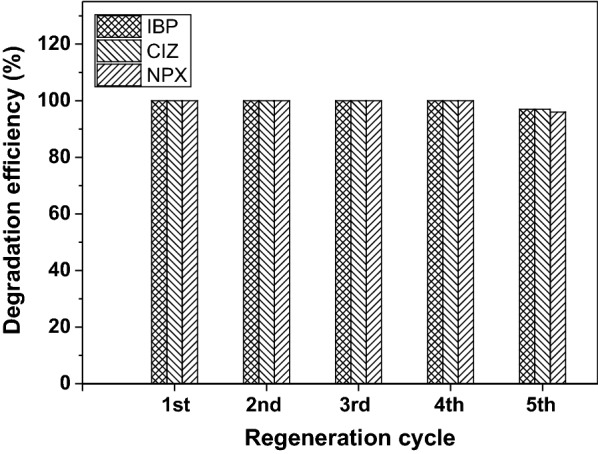
Stability of the composite nanofibers for the photodegradation of IBP, CTZ, and NPX at initial concentration 5 mg/L, drug solution volume of 100 mL, irradiation time of IBP = 120 min, NPX = 40 min, and CIZ = 25 min

The catalyst re-usability examined by cycling experiments under controlled reaction conditions. The reuse of the composite nanofibers is important and has an economic necessity. The PAN-MWCNT/TiO_2_–NH_2_ composite nanofibers were used in sequential photocatalytic process to investigate the durability of the catalytic composite nanofibers. The composite nanofibers washed several times with deionized water and then dried in air after each process, in order to reuse it in other experiments. The Photodegradation efficiency of PAN-MWCNT/TiO_2_–NH_2_ composite nanofibers remained stable during the first four sequential cycles, then decreased by about 7% in the last cycle as shown in Fig. 5.

### Characterization of the composite nanofibers

**Fig. 6:**
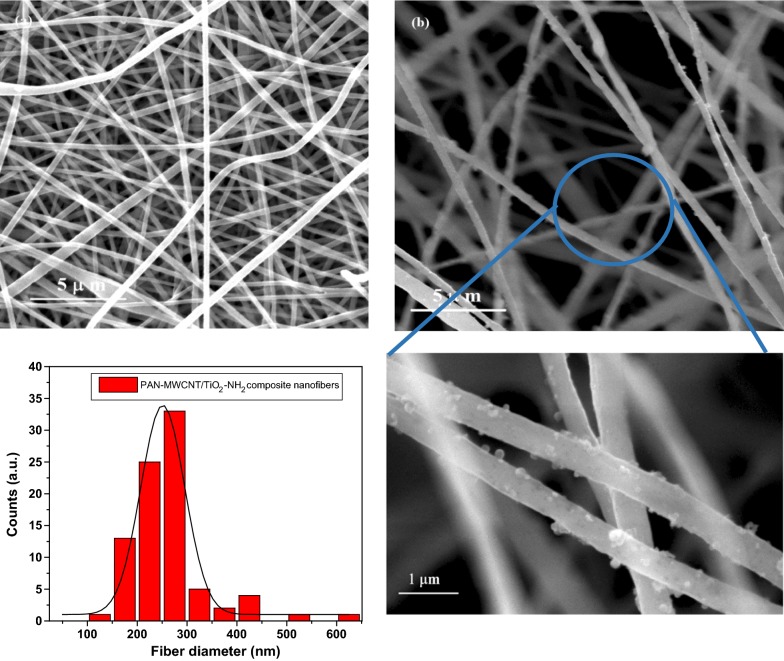
SEM of **a** PAN nanofibers and **b** PAN-MWCNT/TiO_2_–NH_2_ composite nanofibers

Scanning electron microscope (SEM) analysis was performed to investigate the morphological of the electrospun PAN nanofibers and PAN-MWCNT/TiO_2_–NH_2_ composite nanofibers as shown in Fig. 6. It can be seen that the surface morphology of the fabricated composite nanofibers was smooth and continuous with fiber diameters of 251.2 ± 4.6 nm. Figure 6b clearly confirm that the amino functionalized TiO_2_ NPs are attached to the surface of the PAN/MWCNT nanofibers due to the crosslinking process.

**Fig. 7:**
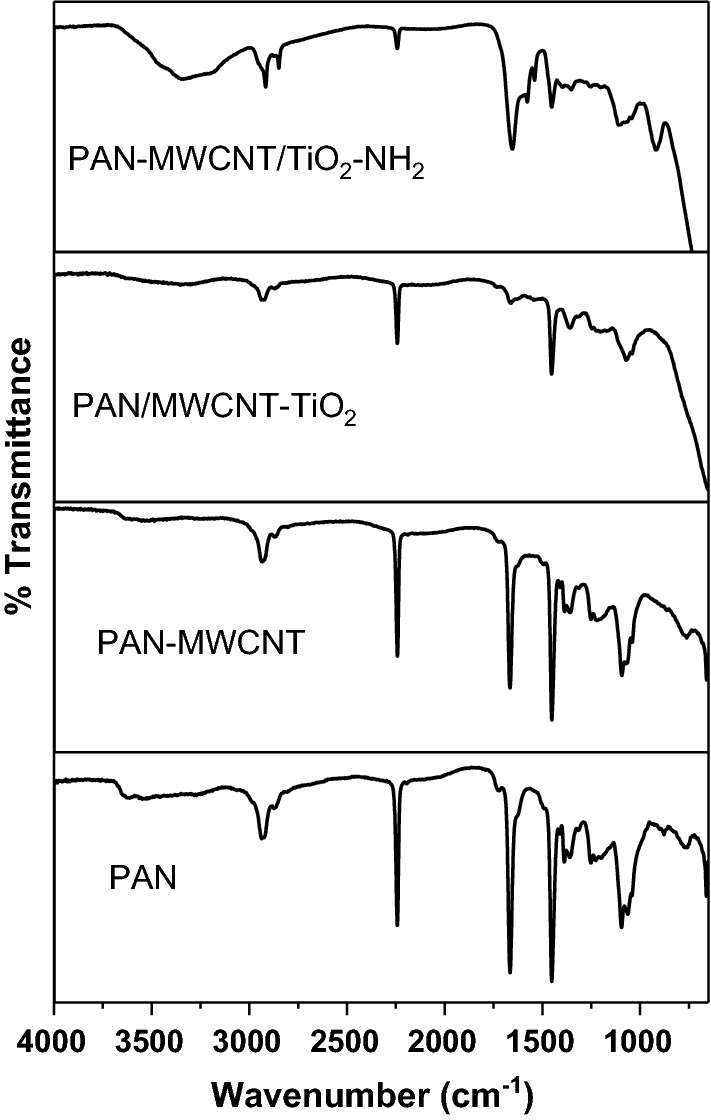
FTIR spectra of PAN, PAN-MWCNT, PAN/MWCNT-TiO_2_, and PAN-MWCNT/TiO_2_–NH_2_ composite nanofibers

The FTIR spectra of PAN, PAN-CNT, PAN/MWCNT-TiO_2_ and PAN-MWCNT/TiO_2_–NH_2_ composite nanofibers are shown in Fig. 7. The spectrum for as prepared PAN nanofibers exhibited characteristic peaks of nitrile at 2242 cm^−1^, carbonyl at 1650 cm^−1^ and C–H stretching at 2940 cm^−1^ [35]. The spectrum for PAN-CNT composite nanofibers exhibit four peaks at 2342, 1700, 3159, and 1290 cm^−1^, which are attributed to nitrile, carbonyl, C–H stretching, and C–O–C stretching vibrations of the MWCNT, indicating the existence of PAN-CNT nanofibers [36]. In addition, the vibrational band at 2000–2500 cm^−1^, is assigned to C ≡ N in the PAN/MWCNT-TiO_2_ composite nanofibers. The bands observed at 3159, 1520, and 1152 cm^−1^ are assigned to the aliphatic C–H bending vibration of the CH_2_ of polymeric chain [37]. The aliphatic C–C band vibrations are observed at 1074 cm^−1^. After the crosslinking of PAN-MWCNT to TiO_2_–NH_2_ NPs, new peaks were observed in the range 3100–3700 cm^−1^ are assigned to N–H and O–H, and the bending vibrations of the amine group NH or NH_2_ observed at 1680 cm^−1^. The band observed at 912 cm^−1^ is assigned to N–O [38].

**Fig. 8:**
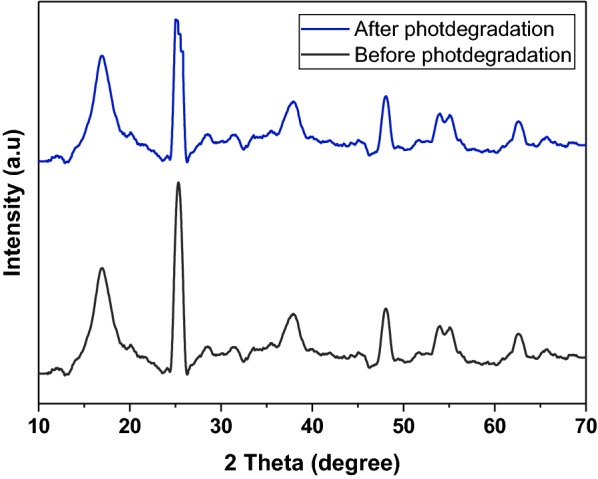
XRD patterns of PAN-CNT/TiO_2_–NH_2_ composite nanofibers before and after the photocatalytic degradation

In addition, XRD patterns were used to identify the crystal phases of the PAN-CNT/TiO_2_–NH_2_ composite nanofibers before and after photocatalysis as shown in Fig. 8. No other impurity diffraction peaks were detected, and the crystal structure is stable after the photocatalytic process. Noticeably, the intensities of the peaks are broad and weak, which indicate that the crystallinity is poor and the crystallite size is small.

## Conclusions

TiO_2_–NH_2_ NPs were successfully cross-linked to the PAN/MWCNT composite nanofibers. This study demonstrated that the fabricated composite nanofibers PAN-MWCNT/TiO_2_–NH_2_ is an effective photocatalyst for the degradation of IBP, NPX, and CIZ in aqueous solutions under UV-light. Operational parameters, such as initial drug concentration, catalyst amount, irradiation time, and solution pH were investigated. It was shown that the complete degradation can be achieved at low drug concentration (5 mg/L), acidic pH = 2, and at a low power intensity of the UV lamp (40 Watt). The complete photodegradation of IBP, NPX, and CIZ required 120, 40, and 25 min, respectively. The stability studies showed that the photodegradation efficiency of PAN-MWCNT/TiO_2_–NH_2_ composite nanofibers remained stable under the experimental conditions studied.

## Abbreviations

IBP: Ibuprofen
NPX: Naproxen
CIZ: Cetirizine
PAN: Polyacrylonitrile
TiO_2_: titanium dioxide
MWCNT: multi-walled carbon nanotube
UV: ultraviolet
APTES: 3-aminopropyltriethoxysilane
GA: Glutaraldehyde
DMF: dimethylformamide
HCI: hydrochloric acid
NaOH: sodium hydroxide
SEM: Scanning Electron Microscopy
FTIR: Fourier transform infrared spectroscopy
XRD: X-ray diffractometry
